# Colonization of long-term care facility residents in three Italian Provinces by multidrug-resistant bacteria

**DOI:** 10.1186/s13756-018-0326-0

**Published:** 2018-03-06

**Authors:** Elisabetta Nucleo, Mariasofia Caltagirone, Vittoria Mattioni Marchetti, Roberto D’Angelo, Elena Fogato, Massimo Confalonieri, Camilla Reboli, Albert March, Ferisa Sleghel, Gertrud Soelva, Elisabetta Pagani, Richard Aschbacher, Roberta Migliavacca, Laura Pagani, AMCLI – GLISTer Group, Laura Pagani, Massimo Confalonieri, Richard Aschbacher, Claudio Farina, Paolo Fazii, Francesco Luzzaro, Pier Giorgio Montanera, ESCMID Study Group Elderly Infections – ESGIE, Roberta Migliavacca

**Affiliations:** 1 Department of Clinical Surgical Diagnostic and Pediatric Sciences, Laboratory of Microbiology and Clinical Microbiology, University of Pavia, Via Brambilla 74, 27100 Pavia, Italy; 2 Laboratory of Clinical Microbiology, ASP “Golgi-Redaelli”, via Bartolomeo d’Alviano 78, 20146 Milan, Italy; 3 O.U. of Microbiology, Azienda Sanitaria Locale di Piacenza, Piacenza, Italy; 4 Geriatric Unit, Comprensorio Sanitario di Bolzano, Bolzano, Italy; 5 Microbiology and Virology Laboratory, Comprensorio Sanitario di Bolzano, Bolzano, Italy

**Keywords:** Long-term care facilities, Multicenter study, ESβL, AmpC, Carbapenemases, MRSA, VRE

## Abstract

**Background:**

Rationale and aims of the study were to compare colonization frequencies with MDR bacteria isolated from LTCF residents in three different Northern Italian regions, to investigate risk factors for colonization and the genotypic characteristics of isolates. The screening included *Enterobacteriaceae* expressing extended-spectrum β-lactamases (ESβLs) and high-level AmpC cephalosporinases, carbapenemase-producing *Enterobacteriaceae*, *Pseudomonas aeruginosa* or *Acinetobacter baumannii*, methicillin-resistant *Staphylococcus aureus* (MRSA) and vancomycin-resistant enterococci (VRE).

**Methods:**

Urine samples and rectal, inguinal, oropharyngeal and nasal swabs were plated on selective agar; resistance genes were sought by PCR and sequencing. Demographic and clinical data were collected.

**Results:**

Among the LTCF residents, 75.0% (78/104), 69.4% (84/121) and 66.1% (76/115) were colonized with at least one of the target organisms in LTCFs located in Milan, Piacenza and Bolzano, respectively. ESβL producers (60.5, 66.1 and 53.0%) were highly predominant, mainly belonging to *Escherichia coli* expressing CTX-M group-1 enzymes. Carbapenemase-producing enterobacteria were found in 7.6, 0.0 and 1.6% of residents; carbapemenase-producing *P. aeruginosa* and *A. baumannii* were also detected. Colonization by MRSA (24.0, 5.7 and 14.8%) and VRE (20.2, 0.8 and 0.8%) was highly variable. Several risk factors for colonization by ESβL-producing *Enterobacteriaceae* and MRSA were found and compared among LTCFs in the three Provinces. Colonization differences among the enrolled LTCFs can be partially explained by variation in risk factors, resident populations and staff/resident ratios, applied hygiene measures and especially the local antibiotic resistance epidemiology.

**Conclusions:**

The widespread diffusion of MDR bacteria in LTCFs within three Italian Provinces confirms that LTCFs are an important reservoir of MDR organisms in Italy and suggests that future efforts should focus on MDR screening, improved implementation of infection control strategies and antibiotic stewardship programs targeting the complex aspects of LTCFs.

**Electronic supplementary material:**

The online version of this article (10.1186/s13756-018-0326-0) contains supplementary material, which is available to authorized users.

## Background

Life expectancy in Italy is rapidly increasing, with present values of 80.1 years for males and 84.7 for females [1]. Due to the ageing population, long-term care facilities (LTCFs), which provide ongoing skilled nursing care to residents and help meet both the medical and non-medical needs of elderly individuals with a chronic illness or disability, play an important role in the Italian healthcare system. Residents in LTCFs have a variety of risk factors for colonization with multidrug-resistant (MDR) bacteria; therefore, these facilities represent reservoirs of: i) *Enterobacteriaceae* expressing extended-spectrum β-lactamases (ESβLs), derepressed/acquired high-level AmpC cephalosporinases or carbapenemases, ii) *Pseudomonas aeruginosa* or *Acinetobacter baumannii* producing carbapenemases and iii) methicillin-resistant *Staphylococcus aureus* (MRSA) and vancomycin-resistant enterococci (VRE) [2–4].

To promote detailed studies of various microbiological aspects related to LTCFs in Italy, the Association of Italian Clinical Microbiologists (Associazione Microbiologi Clinici Italiani; AMCLI) in 2016 has set up a new working group consisting of Clinical Microbiologists (Gruppo di Lavoro per lo Studio delle Infezioni nelle Residenze Sanitarie Assistite e Strutture assimilabili; *GLISTer*); one of the main objectives of this working group is the study of the distribution and prevalence of MDR organisms in Italian LTCFs and therefore a multicenter point-prevalence survey, including the main MDR bacteria as described above, was performed in 2016 on residents of LTCFs, located in three Northern Italian cities.

## Methods

### The aim

Rationale and aims of the study were to compare colonization frequencies with MDR bacteria of LTCF residents in three different Northern Italian cities, located in different Italian regions, and to investigate their genotypic characteristics. Moreover, risk factors for colonization were compared between LTCFs and colonization prevalence was correlated with the local epidemiology of invasive MDR isolates.

### Facilities, patient characteristics and survey design

In October–November 2016, a multicenter point-prevalence screening study was conducted in four LTCFs concerning i) *Enterobacteriaceae* with ESβLs, carbapenemases or high-level AmpCs, ii) *P. aeruginosa* or *A. baumannii* with carbapenemases, iii) MRSA and VRE. The four facilities, located in the Northern Italian Provinces of Milan (*n* = 1), Piacenza (*n* = 2) and Bolzano (*n* = 1), offer high skilled 24 h nursing care.

Although the overall study was performed over a period of 2 months, the sampling interval in each facility lasted for a maximum of 1 week. All residents of the four LTCFs were eligible to participate, and the study was approved by the Ethics Committees of the three referring hospitals; informed written consent was obtained from the residents or, if they were unable to consent, from their relatives.

### Microbiological methods

Sample processing, microbial identification and antibiotic susceptibility testing were carried out in the clinical microbiology laboratories of the referral hospitals. Microbiological methods for the LTCF screening study in Bolzano were previously described [5]. Similar methods were used in the epidemiological studies of Milan and Piacenza LTCFs, with minor modifications.

For the screening of MDR bacteria from LTCF residents in Milan midstream or catheter urine samples were cultured on Oxoid Brilliance™ ESβL plates (Thermo Scientific, UK), applying a 10 μg imipenem (IMP) disc (Oxoid, Thermo Scientific, UK), and on Oxoid Brilliance™ VRE (Thermo Scientific, UK). Inguinal, oropharyngeal and rectal swabs were seeded on Oxoid Brilliance™ ESβL, applying a 10 μg IMP disc, on Oxoid Brilliance™ VRE and on CHROMagar™ MRSA (BD Diagnostics, MD). Nasal swabs were plated on CHROMagar™ MRSA. All plates were incubated at 35 ± 2 °C under aerobic conditions for 24–48 h. Isolate identification and antibiotic susceptibility testing were performed by the BD Phoenix™ System (BD Diagnostics, MD), according to European Committee on Antimicrobial Susceptibility Testing (EUCAST) criteria [6], using PHOENIX NMIC/ID402 for non-urinary Gram-negative bacteria, PHOENIX UNMIC/ID403 for Gram-negative isolates from urine cultures, and PHOENIX PMIC/ID88 for MRSA and VRE. The strains were phenotypically confirmed for β-lactamase production by the ESBL+AMPC Screen Kit and the KPC + MBL Confirm ID Kit (Rosco Diagnostica A/S, Denmark).

Similarly, for screening of MDR bacteria from LTCF residents in Piacenza, midstream or catheter urine samples were seeded on ChromID CPS agar (BioMèrieux, Marcy l’Etoile, France); rectal swabs on ChromID ESBL Agar (BioMèrieux, Marcy l’Etoile, France), on ChromID VRE Agar (BioMèrieux, Marcy l’Etoile, France) and on Mac Conkey agar applying a 10 μg meropenem (MER) disc (Oxoid, Thermo Scientific, UK); nasal swabs on Chapman Agar (Oxoid, Thermo Scientific, UK), on ChromID ESBL and on MacConkey agar applying a 10 μg MER disc; and inguinal swabs on Mannite salt agar (Oxoid, Thermo Scientific, UK). Plates were incubated at 35 ± 2 °C under aerobic conditions for 24–48 h. Isolate identification and antibiotic susceptibility testing were performed using the Vitek 2 System (BioMèrieux, Marcy l’Etoile, France), calibrated against EUCAST criteria [6], with AST-N202 cards (including an ESβL test) for Gram-negative bacteria, AST-P632 cards (with both oxacillin and cefoxitin) for MRSA and AST-P586 cards for VRE. Identification of β-lactamase types was based on Vitek 2 results and on the synergistic effects obtained by the ESβL+AMPC Screen Kit and the KPC + MBL Confirm ID Kit (Rosco Diagnostica A/S, Denmark). VRE were confirmed by vancomycin and teicoplanin Etest strips (BioMèrieux, Marcy l’Etoile, France).

### Molecular characterization of resistance genes

Molecular characterization of all MDR isolates was performed in a common reference laboratory, located at the University of Pavia. Total DNA was extracted by the automated Puro extraction system (DID, Milan, Italy), using the DNA tissue kit, according to manufacturer’s instructions. The presence of ESβL and carbapenemase genes was investigated by PCR, targeting *bla*_CTX-M_-, *bla*_SHV_- *bla*_KPC_-_,_ *bla*_VIM_-, *bla*_IMP_-_,_ *bla*_OXA-48_-, *bla*_NDM_- and *bla*_GES_-type genes, and using published primers and conditions [7–15], summarized in Additional file 1: Table S1. *A. baumannii* isolates were screened for the presence of the following carbapenemase genes: *bla*_OXA-23_-like, *bla*_OXA-24_-like, *bla*_OXA-51_-like and *bla*_OXA-58_-like [16–18]. The presence of IS*Aba1* elements adjacent to *bla*_OXA-51_-like genes was determined as previously described [19]; AmpC genes were detected by a multiplex PCR [20].

Bacterial isolates collected from the LTCF in Milan were screened for *bla*_KPC_-, *bla*_VIM_-_,_ *bla*_OXA-48_- and *bla*_NDM_-type genes by the Cepheid GeneXpert System and confirmed by PCR. Check-MDR CT103 XL array (Check points Health B.V., Wageningen, The Netherlands) has been used to investigate the *bla* gene content of a carbapenem-resistant *P. aeruginosa* strain obtained from an oropharyngeal swab, which tested negative by previous molecular assays.

For gene sequencing, PCR products were purified using the quantum Wizard® SV Gel and PCR Clean-Up System (Promega, Madison, WT, USA) and subjected to double-strand Sanger sequencing. Sequences were analyzed according to the BLAST software [21].

### Statistical analysis

A significance level of *p* ≤ 0.05 was used. In-house physicians reviewed hospital records and, using a standard questionnaire, recorded demographic and clinical data as follows: patient age, gender, length of stay, Barthel immobility score, coma, comorbidities (dementia, urinary incontinence, diabetes, cancer, vascular diseases, chronic obstructive pulmonary disease, decubitus ulcer), presence of infection, antibiotic treatment in the preceding 3 months and the presence of indwelling medical devices. The significance of differences in risk factors and colonization proportions was calculated using the proportion comparison test. Logistic regression analyses were developed to investigate colonization of at least one site with ESβL producers and MRSA as dependent variables, first as univariate and then as multivariate models, including predictors with *p* < 0.05 in the univariate analysis, comprising the specific LTCF of residence, using stepwise logistic selection. Analysis were performed using the Medcalc® software version 15.11.4 (MedCalc software, Ostend, Belgium).

## Results

A variable percentage of LTCF residents, present during the point-prevalence survey in the four LTCFs, agreed to participate: 104/310 (34%) in Milan, 121/326 (37%) in Piacenza (2 LTCFs, with 71/216 and 50/110 participating residents, respectively), and all 115 (100%) residents in the LTCF in Bolzano; no specific LTCF resident selection criteria were used in Milan and Piacenza and resident characteristics of enrolled and not-enrolled residents were similar. The median age of LTCF residents in Milan, Piacenza and Bolzano was 82 years (range: 65–96 years), 86 years (range: 63–102 years) and 77 years (range: 30–94 years) for males, and 90 years (range: 71–102 years), 88 years (69–105 years) and 84 years (24–96 years) for females, respectively. The median length of stay of residents in the LTCFs in Milan, Piacenza and Bolzano was 23 months (range: 1–199 months), 34 months (range: 1–172 months) and 19 months (range: 1–174 months), respectively. Various healthcare staff/resident ratios were found in the LTCFs in Milan (ratio: 0.62; 193/310), Piacenza (ratio: 0.61; 201/326; corresponding to 73/110 and 128/216 in the two enrolled LTCFs, respectively) and Bolzano (ratio: 0.79; 91/115). Demographic and clinical details of the enrolled LTCF residents are summarized in Table 1.

**Table 1:** Demographic and clinical details of LTCF residents from three Italian Provinces

	Milan (M), % (*n* = 104)	Piacenza (P), % (*n* = 121)	Bolzano (B), % (*n* = 115)	Significant differences (*p*-value)
Male sex	30.7	26.4	43.4	M vs. B (0.05); P vs. B (0.006)
Age ≥ 86 years	58.7	60.3	35.6	M vs. B (< 0.001); P vs. B (< 0.001)
Antibiotics in preceding 3 months	24.0	50.4	23.4	M vs. P (< 0.001); P vs. B (< 0.001)
Fluoroquinolones	8.6	7.4	5.2	
Penicillins	2.8	1.6	12.1	M vs. B (0.01); P vs. B (0.001)
Cephalosporins	5.7	24.8	1.7	M vs. P (< 0.001); P vs. B (< 0.001)
Dementia	42.3	79.3	68.7	M vs. P (< 0.001); M vs. B (< 0.001)
Peripheral vascular disease	59.6	47.1	71.3	P vs. B (< 0.001)
Urinary incontinence	74.0	84.3	85.2	M vs. P (0.05); M vs. B (0.04)
Diabetes	19.2	16.5	20.8	
Cancer	8.6	8.2	9.5	
Decubitus ulcer	6.7	5.7	11.3	
Chronic obstructive pulmonary disease	11.5	9.1	18.2	P vs. B (0.04)
Physical disability (Barthel immobility score of 0)	10.4	41.3	67.8	M vs. P (< 0.001); M vs. B (< 0.001); P vs. B (< 0.001)
Coma	0.0	0.0	17.4	M vs. B (< 0.001); P vs. B (< 0.001)
Any medical device	10.5	23.9	38.2	M vs. P (0.009); M vs. B (< 0.001); P vs. B (0.01)
Percutaneous enteral gastrostomy tube	2.8	11.5	20.8	M vs. P (0.01); M vs. B (< 0.001); P vs. B (0.05)
Tracheostomy tube	0.0	1.6	9.5	M vs. B (0.001); P vs. B (0.007)
Urinary catheter	8.6	6.6	18.2	M vs. B (0.04); P vs. B (0.006)
Nasogastric tube	0.0	9.1	1.7	M vs. P (0.001); P vs. B (0.01)
Length of stay in LTCF < 6 months	17.7	8.2	17.3	M vs. P (0.03); P vs. B (0.03)
Hospital admission in previous 12 months, any department	22.3	15.8	38.2	M vs. B (0.01); M vs. P (< 0.001)
				P vs. B (< 0.001)
Geriatrics	0.0	1.6	9.5	M vs. B (*p* = 0.001); P vs. B (*p* = 0.007)
Medicine	4.8	5.7	6.0	
Orthopedics	3.8	3.3	4.3	
Infection	3.8^a^	5.7^b^	0.8^c^	P vs. B (0.03)

^a^Urinary tract infection - UTI (2), respiratory tract infection - RTI (1), infected prosthesis (1)

^b^RTI (6), UTI (1), skin and soft tissue infection (1)

^c^UTI (1)

Isolation frequencies and molecular characterization of the antibiotic resistance determinants are shown in Table 2. A high percentage of LTCF residents were colonized with at least one of the target MDR organisms in Milan (75.0%; 78/104), Piacenza (69.4%; 84/121) and Bolzano (66.1%; 76/115); moreover, many residents from Milan (37.5%; 39/104), Piacenza (19.8%; 24/121) and Bolzano (30.4%; 35/115) were colonized with more than one MDR organism.

**Table 2:** Colonization percentages in residents from LTCFs of three Italian Provinces

	% of LTCF residents colonized with specific resistance phenotype and genotype and significant differences (*p* ≤ 0.05)			
	Milan (*n* = 104)	Piacenza (*n* = 121)	Bolzano (*n* = 115)	Significant differences (*p* ≤ 0.05)
All resistance groups (MRSA; VRE; ESβL-/AmpC-producing enterobacteria; carbapenemase-producing enterobacteria, *Pseudomonas aeruginosa* and *Acinetobacter baumannii*)	75.0	69.4	66.1	
All ESβL-positive enterobacteria	60.5	66.1	53.0	P vs. B (0.04)
*Escherichia coli*, ESβL-positive	48.0	55.3	45.2	
*bla*CTX-M-group-1	33.6	41.3	28.7	P vs. B (0.04)
*bla*CTX-M*-*group-9	6.7	5.7	9.5	
*bla*CTX-M-group, other than 1 or 9	4.8	9.9	0.0	P vs. B (< 0.001)
*Proteus mirabilis*, ESβL-positive	14.4	9.1	7.0	
*bla*CTX-M-group-1	3.8	4.1	0.0	M vs. B (0.04); P vs. B (0.03)
*bla*CTX-M*-*group-9	1.9	0.0	0.0	
*Klebsiella pneumoniae*, ESBL-positive	6.7	5.7	6.1	
*bla*CTX-M-group-1	5.7	4.1	1.7	
*bla*CTX-M*-*group-9	0.9	0.8	0.0	
*bla*CTX-M*-*group, other than 1 or 9	0.0	0.0	2.7	
*Morganella morganii*, ESβL-positive	1.9	1.6	2.6	
*Citrobacter koseri*, ESβL-positive	0.0	3.3	0.8	
*bla*CTX-M-group other than 1 or 9	0.0	3.3	0.0	
*Enterobacter cloacae complex*, ESβL-positive	0.9	0.8	0.0	
*bla*CTX-M-group-1	0.0	0.8	0.0	
*bla*CTX-M-group other than 1 or 9	0.9	0.0	0.0	
*Serratia marcescens*, ESβL-positive	0.0	0.8	0.0	
*bla*CTX-M-group-1*, bla*CTX-M-15-like	0.0	0.8	0.0	
*Providencia stuartii*	1.9	0.0	0.0	
All high-level AmpC-positive enterobacteria	5.7	3.3	25.2	M vs. B (< 0.001); P vs. B (< 0.001)
*Enterobacter cloacae complex*, high-level AmpC	0.0	0.8	0.0	
*Morganella morganii*, high-level AmpC	3.8	0.8	24.3	M vs. B (< 0.001); P vs. B (< 0.001)
*bla*DHA-type	3.8	0.8	8.7	P vs. B (0.004)
*Citrobacter freundii*, high-level AmpC	0.0	0.8	0.0	
*Proteus mirabilis*, high-level AmpC	1.9	0.0	0.8	
*bla*CMY-type	0.0	0.0	0.8	
*Serratia marcescens,* high-level AmpC	0.0	0.8	0.0	
*Providencia rustigianii,* high-level AmpC	0.9	0.0	0.0	
All carbapenemase-positive enterobacteria	7.6	0.0	1.6	M vs. P (0.002); M vs. B (0.03)
*Klebsiella pneumoniae, bla*KPC-type	6.7	0.0	0.0	M vs. P (0.004); M vs. B (0.05)
*Escherichia coli, bla*VIM-1	0.0	0.0	0.8	
*Enterobacter cloacae complex, bla*VIM-1	0.9	0.0	0.0	
*Citrobacter amalonaticus, bla*VIM-1	0.0	0.0	0.8	
Carbapenemase-positive *Pseudomonas aeruginosa*	0.0	1.6	0.0	
*bla*VIM-type	0.0	0.8	0.0	
*bla*GES-5	0.0	0.8	0.0	
Carbapenemase-positive *Acinetobacter baumannii*	1.9	5.8	0.0	P vs. B (0.009)
*bla*OXA-23-like	1.9	5.8	0.0	P vs. B (0.009)
MRSA	24.0	5.7	14.8	M vs. P (< 0.001); P vs. B (0.02)
VRE	20.2^a^	0.8^a^	0.8^b^	M vs. P (< 0.001); M vs. B (< 0.001)

Notes: ^a^*Enterococcus faecalis*; ^b^*Enterococcus faecium*

ESβL-producing *E. coli* expressing *bla*_CTX-M_-like genes were highly predominant in Milan (80.4%), Piacenza (97.0%) and Bolzano (80.3%) and CTX-M-type determinants were also identified in *Proteus mirabilis*, *Klebsiella pneumoniae*, *Citrobacter koseri*, *Enterobacter cloacae complex* and *Serratia marcescens*. Most *bla*_CTX-M-_ genes belonged to group-1 (72.4%), followed by group-9 (14.8%) and other groups (12.8%). A *bla*_BEL_-like gene was detected in a *P. aeruginosa* strain from the LTCF in Milan.

In total, ten carbapenemase-producing *Enterobacteriaceae* were detected: *n* = 7 KPC-producing *K. pneumoniae* and *n* = 1 VIM-1-producing *E. cloacae complex* were isolated from LTCF residents in Milan, and *n* = 2 VIM-1 producers (one *E. coli* and one *Citrobacter amalonaticus*) from residents in Bolzano. Two carbapenemase-positive *P. aeruginosa* were isolated from LTCF residents in Piacenza: in one case a *bla*_GES-5_ and in the other a *bla*_VIM_-like gene were identified. Moreover, two *P. aeruginosa* isolates collected in Milan and Piacenza presented a *bla*_GES-1_ ESβL. Nine *bla*_OXA-23_-positive *A. baumannii* were isolated from two and seven LTCF residents in Milan and Piacenza, respectively.

MRSA strains were most frequently isolated from LTCF residents in Milan and Bolzano, whereas VRE isolates were highly prevalent in Milan (*n* = 21 *Enterococcus faecalis*), but rare in Piacenza (*n* = 1 *E. faecalis*) and Bolzano (*n* = 1 *Enterococcus faecium*).

Colonization of LTCF residents with ESβL-producing enterobacteria and MRSA was associated with several risk factors in univariate and multivariate analysis (Table 3). In multivariate analysis, the LTCF of residence was an independent risk factor for ESβL (*p* ≤ 0.03 for all comparisons, except *p* = 0.53 for the comparison of Milan vs. Piacenza) and MRSA (*p* ≤ 0.02 for all comparisons) colonization. Risk factors for MRSA colonization were also associated with resident’s gender; for the following risk factors significant differences between male (*n* = 226) and female (*n* = 114) residents were found: age > 85 years (M: 34.5%; F: 20.4%; *p* < 0.001), hospitalization within the previous 12 months (M: 35.0%; F: 20.4%; *p* = 0.03), administration of any antibiotic within the previous 3 months (M: 40.3%; F: 29.6%; *p* = 0.04) and coma (M: 10.5%; F: 3.5%; *p* = 0.009).

**Table 3:** Resident’s risk factors for ESβL and MRSA colonization (cumulative data: Milan, Piacenza, Bolzano)

			Univariate analysis		Multivariate analysis				Univariate analysis		Multivariate analysis	
	ESBL, % (*n* = 203)	No ESBL, % (*n* = 137)	OR (CI 95%)	p	OR (CI 95%)	p	MRSA, % (*n* = 45)	No MRSA, % (*n* = 295)	OR (CI 95%)	p	OR (CI 95%)	p
Male sex	34.9	31.3	1.17 (0.74–1.86)	0.49			*51.1*	*30.8*	*2.34 (1.24–4.42)*	*0.008*	*2.31 (1.16–4.59)*	*0.01*
Age ≥ 86 years	52.7	49.2	1.15 (0.74–1.78)	0.53			39.0	53.0	0.56 (0.29–1.10)	0.09		
Antibiotics in preceding 3 months	*39.9*	*23.3*	*2.17 (1.34–3.54)*	*0.001*	*1.74 (1.02–2.98)*	*0.04*	37.7	32.5	1.25 (0.65–2.41)	0.48		
Fluoroquinolones	7.8	5.8	1.38 (0.57–3.32)	0.47			*15.5*	*5.7*	*3.01 (1.17–7.73)*	*0.02*	*3.59 (1.26–10.25)*	*0.01*
Penicillins	7.3	2.9	2.65 (0.86–8.17)	0.09			11.1	4.7	2.50 (0.85–7.34)	0.09		
Cephalosporins	14.2	6.5	2.37 (1.08–5.18)	0.03			4.4	12.2	0.33 (0.07–1.44)	0.14		
Dementia	63.0	66.4	0.86 (0.54–1.36)	0.52			62.2	64.7	0.89 (0.47–1.71)	0.74		
Peripheral vascular disease	62.5	59.1	1.15 (0.74–1.80)	0.52			62.2	61.0	1.05 (0.55–2.00)	0.87		
Urinary incontinence	83.2	78.8	1.33 (0.77–2.31)	0.30			82.2	81.3	1.06 (0.46–2.40)	0.89		
Diabetes	18.7	18.9	0.98 (0.56–1,71)	0.30			26.6	17.6	1.69 (0.82–3.51)	0.15		
Cancer	*11.8*	*4.3*	*2.92 (1.16–7.36)*	*0.02*	*3.47 (1.32–9.16)*	*0.01*	4.4	9.5	0.44 (0.10–1.93)	0.27		
Decubitus ulcer	9.8	5.1	2.03 (0.83–4.94)	0.12			6.6	8.1	0.80 (0.23–2.79)	0.73		
Chronic obstructive pulmonary disease	11.8	14.6	0.78 (0.41–1.48)	0.45			15.5	12.5	1.28 (0.53–3.08)	0.57		
Physical disability (Barthel immobility score of 0)	*47.7*	*32.3*	*1.91 (1.21–3.02)*	*0.005*	*2.10 (1.15–3.83)*	*0.01*	37.7	41.0	0.87 (0.45–1.66)	0.67		
Coma	6.9	4.3	1.61 (0.60–4.31)	0.33			6.6	5.7	1.16 (0.32–4.15)	0.81		
Any medical device	*32.5*	*13.1*	*3.18 (1.79–5.66)*	*< 0.001*	*2.81 (1.44–5.47)*	*0.002*	33.3	23.3	1.63 (0.83–3.21)	0.15		
Percutaneous enteral gastrostomy tube	15.7	6.5	2.66 (1.22–5.77)	0.01			11.1	12.2	0.89 (0.33–2.42)	0.83		
Tracheostomy tube	4.9	2.1	2.31 (0.62–8.56)	0.21			4.4	3.7	1.20 (0.25–5.60)	0.81		
Urinary catheter	15.7	4.3	4.08 (1.66–10.06)	0.002			*20.0*	*9.8*	*2.29 (1.00–5.23)*	*0.04*	*2.61 (0.06–6.43)*	*0.03*
Nasogastric tube	5.9	0.7	8.54 (1.09–66.49)	0.04			4.4	3.7	1.20 (0.25–5.60)	0.81		
Length of stay in LTCF < 6 months	15.6	11.9	1.36 (0.71–2.61)	0.34			16.6	13.7	1.25 (0.51–3.00)	0.61		
Hospital admission in previous 12 months	24.2	27.0	0.87 (0.53–1.43)	0.58			*37.7*	*23.4*	*1.97 (1.02–3.81)*	*0.04*		
Infection	5.4	2.9	1.90 (0.59–6.11)	0.27			8.8	3.7	2.51 (0.76–8.28)	0.12		

ND: not determined; factors included in multivariate analysis are in italics. For multivariate analysis only significant values are shown

## Discussion

The study evaluated the degree of colonization with drug-resistant bacteria among residents of LTCFs located in three Northern Italian Provinces, finding high colonization of residents in Milan (75.0%), Piacenza (69.4%) and Bolzano (66.1%). Many residents had more than one target organism, underscoring the role of LTCFs as a reservoir for these isolates [2–4].

Colonization of LTCF residents with ESβL-producing enterobacteria was highly prevalent in all the surveyed LTCFs (60.5% in Milan, 66.1% in Piacenza and 53.0% in Bolzano), and group-1 CTX-M-type enzymes were highly predominant, especially in *E. coli* (80–97% of isolates). Notably, about 82% of *K. pneumoniae* and 32% of *P. mirabilis* isolates also harbored a *bla*_CTX-M_-type gene. In the same Bolzano LTCF, here screened for ESβL-producing enterobacteria, high colonization percentages, equal to 64.0 and 49.0%, were previously found in 2008 [22] and 2012 [23], respectively; the latter survey also screened a second LTCF in the Province of Bolzano, showing a colonization prevalence of 56.0%. In an Italian study carried out in 2006, a colonization prevalence of 54.0% was found in LTCF residents bearing a urinary catheter [24], while a more recent multicenter study, performed in 2015 and involving 12 Italian LTCFs, reported a mean ESβL colonization of 57.3% (range: 32.8–81.5%) [25]. In all these Italian studies, CTX-M enzymes were the predominantly produced ESβLs. The high ESβL colonization rates of > 50% in Italian LTCF residents are paralleled by high ESβL prevalence in invasive *E. coli* isolates [26]. Generally, ESβL carriage in most European countries is strikingly lower than that found in Italy [4], with exceptions reported from Ireland [27, 28] and Portugal [29].

In our screening study, high-level AmpC-producing *Enterobacteriaceae* were rarely isolated in LTCF residents in Milan and Piacenza, but 24.3% of LTCF residents in Bolzano were colonized by *M. morganii* expressing a high-level DHA-AmpC phenotype; *bla*_DHA_-type genes in LTCF isolates have previously been found in a few *E. coli* and *K. pneumoniae* strains from Korea [30], but to our knowledge have not yet been reported in Italian LTCFs.

Carbapenemase-producing enterobacteria were not found in LTCF residents in Piacenza, rarely in Bolzano (1.6%) and more frequently in Milan (7.6%). As found in previous studies of carbapenemase-producing *Enterobacteriaceae* from Bolzano [22, 23, 31], the VIM-1-producing *E. coli* and *C. amalonaticus* isolates from residents in this study were also positive for *bla*_SHV-12_. In the present study, all carbapenemase producers from Milan, except an *E. cloacae complex* isolate expressing a *bla*_VIM-1_ gene, had KPC-type enzymes; similar results have been reported by other Italian studies in LTCF residents [25, 32, 33]. Carbapenemase-producing enterobacteria, especially KPC-producing *K. pneumoniae*, are epidemically spread in Italy [34] and the emergence of this MDR phenotype in LTCFs is worrying, expanding the reservoir of this health care threat. Nevertheless, as previously summarized [4], carbapenemase-producing *Enterobacteriaceae* are still rare in Italian LTCF residents; the reasons are probably multifactorial, comprising clinical characteristics of the enrolled residents [35] and the low carbapenem selective pressure in LTCFs. On average, only 1.1% of residents enrolled in our screening study received carbapenems within the previous 3 months (data not shown). Nevertheless, a carbapenemase-producing enterobacteria prevalence of 7.6% (mainly KPC-producing *K. pneumoniae*), reported here for the LTCF in Milan, gives rise to concern and has to be addressed by future hygiene and antibiotic stewardship measures.

This study shows the emergence of carbapenemase-producing *P. aeruginosa* in LTCF residents in Piacenza, identifying single isolates with *bla*_VIM_-type and *bla*_GES-5_ determinants. *P. aeruginosa* expressing *bla*_VIM_-type determinants is widely spread in Italy [36], and an outbreak of GES-5-producing *P. aeruginosa* was reported from a LTCF in Japan [37]. Moreover, the ESβL genes *bla*_GES-1_ and *bla*_BEL_-like were found in two and one *P. aeruginosa* isolates, respectively; the latter rarely detected β-lactamase was previously recovered in *P. aeruginosa* strains from Belgium [18]. *A. baumannii* producing OXA-23 carbapenemases have an epidemic diffusion in Italy [38], reflected in the present study by the isolation of this resistance type from LTCF residents in Milan (1.9%) and Piacenza (5.8%).

MRSA colonization prevalence here reported ranged widely in the surveyed LTCFs (5.7, 14.8 and 24.0% in Milan, Piacenza and Bolzano, respectively), similar to other Italian studies [25, 39, 40]. Varying MRSA colonization prevalence, ranging from close to zero up to levels higher than 37%, has been reported in European studies [4].

Colonization by VRE in the present study was highly variable, ranging from 0.8 to 20.2%. VRE-carriage in European LTCF residents was found to be low, ranging from 0.0–3% [28, 41, 42].

For *Enterobacteriaceae* significant differences in colonization frequencies of LTCF residents were found: i) for CTX-M-type ESβL-producing *E. coli* between Piacenza (highest prevalence) and Bolzano, ii) for high-level AmpC-producing *M. morganii* (highest prevalence in Bolzano), iii) for carbapenemase producers, with highest prevalence in Milan, iv) for carbapenemase-producing *A. baumannii*, showing highest prevalence in Piacenza, and v) for MRSA and VRE, most prevalent in Milan. Therefore, no clear picture of general colonization differences can be deduced from overall colonization prevalence data.

A variety of risk factors for MRSA and ESβL colonization have previously been reported [4]; many of these have also been analyzed in the present survey. Interestingly, male residents carried a more than double risk for MRSA carriage when compared with female residents, probably because of the higher frequencies of other risk factors in males (administration of any antibiotic within the previous 3 months, hospitalization within the previous 12 months and coma), predisposing men rather than women to MRSA acquisition. Moreover, in our study the trend for an inverse correlation (*p* = 0.09) between age > 85 years and MRSA prevalence was associated with a significantly lower percentage of male residents > 85 years, compared to females; similar results have been found by other authors [43]. In the present survey, administration of cephalosporins during the previous 3 months resulted to be an independent risk factor for ESβL colonization; the LTCFs in Piacenza registered the highest consumption of cephalosporins, correlating with highest ESβL prevalence in LTCF residents from Piacenza. Other independent risk factors for ESβL colonization were physical disability, the presence of any invasive medical device and cancer. Whereas no significant differences were found between residents in the three Provinces for cancer as risk factor, physical disability and the presence of any medical device showed highest prevalence in the LTCF in Bolzano; nonetheless, LTCF residents in Bolzano had the lowest ESβL prevalence in the present screening study.

Therefore, further factors may have contributed to the observed differences, comprising staff/resident ratio and practiced hygiene and infection control measures [44]. The LTCF in Bolzano showed the highest staff/resident ratio, and understaffing has been shown to be a risk factor for colonization of LTCF residents by MDR organisms [2]. All of the surveyed LTCFs in the present study follow hygiene, infection prevention and control measures according to guidelines of The Society for Healthcare Epidemiology of America (SHEA) and The Association for Professionals in Infection Control and Epidemiology (APIC) [45]. Nonetheless, the Bolzano LTCF had introduced enforced hygiene measures, according to the World Health Organization guidelines [46], after the 2008 screening study, showing an ESβL colonization prevalence of 64.0% in LTCF residents [22]; colonization frequency decreased significantly to 49.0% (*p* = 0.02) in 2012 [23], arriving at a slightly higher percentage of 53.0% in 2016, but other factors such as changed case mixes and risk factors may also have contributed to this decrease in ESβL prevalence [23].

Significant differences in antibiotic resistance epidemiology of blood culture isolates, used as a proxy for the general local antibiotic resistance epidemiology, were registered, as derived from European Antimicrobial Resistance Surveillance Network (EARS-Net) data for 2016 [26]. Specifically, we found the following antibiotic resistance data referred to the geographic regions of Milan, Piacenza and Bolzano, respectively: *E. coli* third generation cephalosporin-resistant: 22.1% (29/131), 29.4% (71/259) and 17.8% (56/314); *K. pneumoniae* carbapenem-resistant: 29.2% (7/24), 13.5% (10/74) and 6.2% (4/64); *A. baumannii* carbapenem-resistant: 50.0% (1/2,) 100.0% (24/24) and 0.0% (0/2); MRSA: 36.0% (18/50), 49.7% (82/165) and 14.6% (20/137); *E. faecalis* VRE: 0.0% (0/20), 2.4% (2/83) and 0.0% (0/41); *E. faecium* VRE: 10.0% (1/10), 22.2% (6/27) and 8.0% (2/25). This data for blood culture isolates, compared with our LTCF screening data, correlates well for ESβL-producing *E. coli*, carbapenem-resistant *K. pneumoniae* and *A. baumannii*; on the other hand, no correlation for MRSA and VRE can be derived. Patient transfer between acute-care facilities and LTCFs contribute to the diffusion of MDR organisms in both settings; such bi-directional movement of MDR bacteria, related to acute systemic infections, might be more significant for *Enterobacteriaceae* and *A. baumannii* than for MRSA and VRE.

Moreover, the snapshot approach used in this study might lead to the sudden increase in prevalence of a specific resistance phenotype, as shown for high-level AmpC-producing *M. morganii* detected in 2016 from Bolzano LTCF residents [5], which could be a transient phenomenon. Similarly, the high prevalence of VRE in LTCF residents from Milan could be due to a transitory local epidemic event.

Finally, the local circulation of highly transmissible clones, for example ESβL-producing *E. coli*, KPC-producing *K. pneumoniae* and OXA-23-producing *A. baumannii* could contribute to the explanation of the here reported screening results [38, 47].

This study has some limitations. First, it has been done in only four LTCFs, located in three different Provinces in Northern Italy, and therefore data may not be extrapolated to other Italian LTCFs with differing characteristics. Second, the number of LTCF residents participating in the study was variable, ranging from 34% in Milan up to 100% in Bolzano. Third, we did not use an enrichment step during the laboratory analysis; this limitation is partially compensated by using 4–5 different specimen types for the screening of MDR bacteria. Fourth, different sample types, types of media and laboratory methodologies have been used in the three laboratories processing the samples from the different LTCFs. Fifth, molecular characterization and typing of isolates in the 2016 study was limited, not including pulsed-field gel electrophoresis (PFGE) and sequence typing (ST) of isolates and therefore not permitting the identification of epidemic clusters. Finally, screening of healthcare workers has been done only in one of the enrolled LTCFs [5], but not in the other surveyed facilities. Despite these limitations, the strength of our study is the comparison of colonization prevalence between LTCFs located in three different Provinces, comparing it also with differences in risk factors for colonization and in the local epidemiology of invasive isolates.

## Conclusions

We performed a multicenter point-prevalence study in LTCFs located in three different Provinces in Northern Italy and found high colonization prevalence of LTCF residents for MDR organisms, especially ESβL-producing *E. coli*. Variability between the different facilities was noticeable also for other MDR organisms. Differences can be partially explained by i) differences in risk factors for colonization by MDR organisms, ii) changes in resident populations and staff/resident ratios, iii) applied hygiene measures and iv) differences in the local epidemiology of antibiotic resistance of clinical isolates. This widespread diffusion of MDR bacteria in LTCFs of three Italian Provinces confirms that these healthcare facilities are an important reservoir for MDR organisms. Future efforts should focus on screening activities, infection control strategies tailored on the complex aspects of LTCFs and implementation of antibiotic stewardship programs.

## Additional file

Additional file 1: **Table S1.** Oligonucleotides used for PCR and sequencing. (DOCX 17 kb)

## Abbreviations

AMCLI: Association of Italian Clinical Microbiologist
APIC: The Association for Professionals in Infection Control and Epidemiology
CTX-M: Cefotaximase-Munich type Extended-Spectrum β-Lactamase
EARS-Net: European Antimicrobial Resistance Surveillance Network
ESβL: Extended-Spectrum β-Lactamase
EUCAST: European Committee on Antimicrobial Susceptibility Testing
GLISTer: Gruppo di Lavoro per lo Studio delle Infezioni nelle Residenze Sanitarie Assistite e Strutture Assimilabili
KPC: *Klebsiella Pneumoniae* Carbapenemase
LTCF: Long-Term Care Facility
MBL: Metallo-β-Lactamase
MDR: Multidrug-Resistant
MRSA: Methicillin-Resistant *Staphylococcus aureus*
PCR: Polymerase Chain Reaction
PFGE: Pulsed-Field Gel Electrophoresis
SHEA: The Society for Healthcare Epidemiology of America
ST: Sequence Typing
VIM: Verona Integron-Encoded Metallo-β-Lactamase
VRE: Vancomycin-Resistant Enterococci
