# Supplementary material for: Colonization of long-term care facility residents in three Italian Provinces by multidrug-resistant bacteria
Source: Antimicrob Resist Infect Control. 2018 Mar 6;7:33. doi: 10.1186/s13756-018-0326-0 (PMC5839059; doi:10.1186/s13756-018-0326-0)
Supplement: Supplementary file 1 — Table S1. Oligonucleotides used for PCR and sequencing. (DOCX 17 kb) [file 13756_2018_326_MOESM1_ESM.docx]

| **Additional file 1: Table S1** Oligonucleotides used for PCR and sequencing. | | | | |
| --- | --- | --- | --- | --- |
| **Genes** | **Primer sequences** | **T*a* ^1^** | **Fragment sizes** | **References** |
| ***bla*_CTX-M-_type** | FW 5’-ATGTGCAGYACCAGTAARGT-3’  REV 5’-TGGGTRAARTARGTSACCAGA-3’ | 50°C | 593 bp | Pagani et al. 2003 [7] |
| ***bla*_CTX-M-1-_ group** | FW 5’-GGTTAAAAAATCACTGCGTC -3’  REV 5’- TTGGTGACGATTTTAGCCGC-3’ | 50°C | 1000 bp | Eckert et al. 2006 [8] |
| ***bla*_CTX-M-9_- group** | FW 5’- ATGGTGACAAAGAGAGAGTGCA-3’  REV 5’- CCCTTCGGCGATGATTCTC-3’ | 56°C | 835 bp | Eckert et al. 2006 [8] |
| ***bla*_SHV_-type** | FW 5′-GCCCGGGTTATTCTTATTTGTCGC-3’  REV 5′-TCTTTCCGATGCCGCCGCCAGTCA-3' | 60°C | 900 bp | Perilli et al. 2002 [9] |
| ***bla*_TEM_-type** | FW 5´-ATGAGTATTCAACATTTCCG-3’  REV 5’-CTGACAGTTACCAATGCTTA-3’ | 59°C | 800 bp | Rasheed JK et al. 1997 [10] |
| ***bla*_KPC_-type** | FW 5´-TGTCACTGTATCGCCGTC-3’  REV 5’-CTCAGTGCTCTACAGAAAACC-3’ | 55°C | 1000 bp | Yigit et al. 2001 [11] |
| ***bla*_OXA-48_** | FW: 5’-TTGGTGGCATCGATTATCGG-3’  REV: 5’-GAGCACTTCTTTTGTG ATGGC-3’ | 52°C | 743 bp | Poirel et al. 2011 [12] |
| ***bla*_VIM_-type**  ***bla*_IMP_*-*type**  ***bla*_NDM_*-*type**  ***bla*_GES_*-*type**  ***bla*_OXA-23_-like**  ***bla*_OXA-58_*-*like**  ***bla*_OXA-24_*_-_*like**  ***ISAba1*+*bla*_OXA-51_-like** | FW 5’-CAGATTGCCGATGGTGTTTGG-3’  REV 5’-AGGTGGGCC ATTCAGCCAGA-3’  FW 5’-GGAATAGAGTGGCTTAATTCTC-3’  REV 5’-GTGATGCGTCYCCAAYTTCACT-3’  FW 5’- GGTTTGGCGATCTGGTTTTC-3’  REV 5’- CGGAATGGCTCATCACGATC-3’  FW 5’- ATGCGCTTCATTCACGCAC-3’  REV 5’-CTATTTGTCCGTGCTCAGG-3’  FW 5’-GAT CGG ATT GGA GAA CCA GA-3’  REV 5’-ATT TCT GAC CGC ATT TCC AT-3’  FW 5’-AAG TAT TGG GGC TTG TGC TG-3’  REV 5’-CCC CTC TGC GCT CTA CAT AC-3’  FW 5’-GGT TAG TTG GCC CCC TTA AA-3’  REV 5’- AGT TGA GCG AAA AGG GGATT-3’  FW 5’-CACGAATGCAGAAGTTG-3’  REV 5’-CGAACAGAGCTAGRTATTC-3’ | 55°C  50°C  52°C  61°C  51°C  51°C  51°C  58 °C | 523 bp  361 bp  621 bp  860 bp  501 bp  599 bp  246 bp  1200 bp | Migliavacca et al. 2002 [13]  Lagatolla et al. 2004 [14]  Poirel et al. 2011 [12]  Poirel et al. 2000 [15]  Woodford N et al. 2006 [16]  Woodford N et al. 2006 [16]  Woodford N et al. 2006 [16]  Turton et al. 2006 [19] |
| **^1^**T*a*: annealing temperature. | | | | |
